# European Achondroplasia Forum guiding principles for the detection and management of foramen magnum stenosis

**DOI:** 10.1186/s13023-023-02795-2

**Published:** 2023-07-27

**Authors:** Melita Irving, Moeenaldeen AlSayed, Paul Arundel, Geneviève Baujat, Tawfeg Ben-Omran, Silvio Boero, Valérie Cormier-Daire, Svein Fredwall, Encarna Guillen-Navarro, Heike Hoyer-Kuhn, Philip Kunkel, Christian Lampe, Mohamad Maghnie, Klaus Mohnike, Geert Mortier, Sérgio B. Sousa

**Affiliations:** 1 Department of Clinical Genetics, Guy’s and St Thomas’ NHS Foundation Trust, London, UK; 2 Department of Medical Genomics, King Faisal Specialist Hospital and Research Center and Faculty of Medicine, Alfaisal University, Riyadh, Kingdom of Saudi Arabia; 3 Department of Metabolic Bone Disease, Sheffield Children’s NHS Foundation Trust, Sheffield, UK; 4 Centre of Reference for Constitutional Bone Diseases (MOC), Department of Clinical Genetics, Paris Centre University, Imagine Institute, Necker-Enfants Malades Hospital, Paris, France; 5 Division of Genetics and Genomic Medicine, Sidra Medicine & Hamad Medical Corporation, Doha, Qatar; 6 Pediatric Orthopaedic and Traumatology Unit, Istituto Giannina Gaslini, Genoa, Italy; 7 Centre of Reference for Constitutional Bone Diseases (MOC), Department of Clinical Genetics, Paris Centre University, INSERM UMR 1163, Imagine Institute, Necker-Enfants Malades Hospital, Paris, France; 8 TRS National Resource Centre for Rare Disorders, Sunnaas Rehabilitation Hospital, Nesodden, Norway; 9 Medical Genetics Section, Department of Pediatrics, Virgen de la Arrixaca University Clinical Hospital, IMIB-Pascual Parrilla, University of Murcia-UMU, Murcia; CIBERER-ISCIII, Madrid, Spain; 10 Children’s Hospital, University Cologne, Cologne, Germany; 11 Department of Neurosurgery, University Medical Centre Mannheim, Medical Faculty Mannheim, Heidelberg University, Mannheim, Germany; 12 Clinic of Neuropediatrics, Epileptology and Social Pediatrics, University Hospital Giessen and Marburg, Giessen, Germany; 13 Department of Pediatrics, IRCCS Istituto Giannina Gaslini, Genova, 16147 Italy; 14 Department of Neuroscience, Rehabilitation, Ophthalmology, Genetics, Maternal and Child Health, University of Genova, Genova, 16147 Italy; 15 Central German Competence Network for Rare Diseases (ZSE), Universitätskinderklinik, Otto-von-Guericke Universität, Magdeburg, Germany; 16 Department of Medical Genetics and Centre for Rare Diseases, Centre of Human Genetics, KU Leuven, Leuven, Belgium; 17 Medical Genetics Unit, Hospital Pediátrico, Centro Hospitalar e Universitário de Coimbra; and University Clinic of Genetics, Faculty of Medicine, Universidade de Coimbra, Coimbra, Portugal

**Keywords:** Achondroplasia, European Achondroplasia Forum, Foramen Magnum Stenosis, Guiding principles, Detection, Management, Recommendations

## Abstract

Foramen magnum stenosis is a serious, and potentially life-threatening complication of achondroplasia. The foramen magnum is smaller in infants with achondroplasia, compared with the general population, and both restricted growth in the first 2 years and premature closure of skull plate synchondroses can contribute to narrowing. Narrowing of the foramen magnum can lead to compression of the brainstem and spinal cord, and result in sleep apnoea and sudden death. There is a lack of clarity in the literature on the timing of regular monitoring for foramen magnum stenosis, which assessments should be carried out and when regular screening should be ceased. The European Achondroplasia Forum (EAF) is a group of clinicians and patient advocates, representative of the achondroplasia community. Members of the EAF Steering Committee were invited to submit suggestions for guiding principles for the detection and management of foramen magnum stenosis, which were collated and discussed at an open workshop. Each principle was scrutinised for content and wording, and anonymous voting held to pass the principle and vote on the level of agreement. A total of six guiding principles were developed which incorporate routine clinical monitoring of infants and young children, timing of routine MRI screening, referral of suspected foramen magnum stenosis to a neurosurgeon, the combination of assessments to inform the decision to decompress the foramen magnum, joint decision making to proceed with decompression, and management of older children in whom previously undetected foramen magnum stenosis is identified. All principles achieved the ≥ 75% majority needed to pass (range 89–100%), with high levels of agreement (range 7.6–8.9). By developing guiding principles for the detection and management of foramen magnum stenosis, the EAF aim to enable infants and young children to receive optimal monitoring for this potentially life-threatening complication.

## Background

**Fig. 1:**
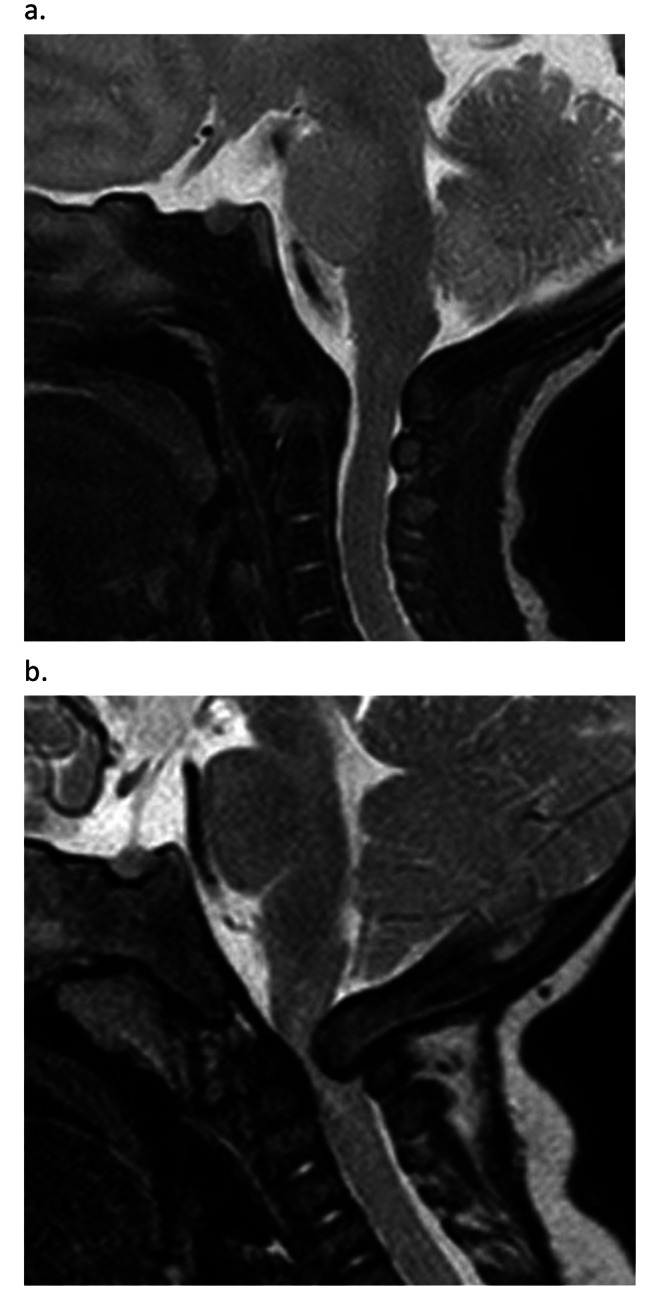
A case of an asymptomatic infant with achondroplasia a) as a newborn and b) at 4 months with neurological signs, foramen magnum stenosis and spinal cord compression, Fornarino Grade 3 [5]

Achondroplasia is the most common form of skeletal dysplasia, with a prevalence of 3.72–4.6 per 100,000 births [1]. It is a complex and multifaceted condition with complications that occur at all stages of the life course, including, among others, foramen magnum stenosis (FMS), otitis media, genu varum, spinal complications, pain and sleep apnoea [2, 3]. The most severe and life-threatening of the complications observed in infancy and early childhood, is foramen magnum stenosis. The foramen magnum is situated in the occipital bone, within the posterior cranial fossa, and is traversed by the medulla oblongata, meninges, vertebral arteries, anterior and posterior spinal arteries, dural veins and the spinal roots of accessory nerves [4, 5]. In babies with achondroplasia, the foramen magnum is significantly smaller than in individuals of average stature, and is further impacted by restricted growth in the first 2 years of life, and premature closure of the skull base synchondroses [6, 7]. This can result in a foramen magnum that is small and misshapen leading to compression of the brainstem and spinal cord (Fig. 1). There is evidence that this compression is linked to sleep apnoea and sudden death in infants [6, 8]. Rates of mortality as a result of FMS differ in the literature, ranging from 2 to 7.5%, [3, 9] however there is agreement that as a consequence there is an increase in mortality in infants with achondroplasia compared to the general population [9–11].

Infants and younger children are at greater risk of FMS than older children or adults [12]. However, there is no specific recommendation in the American Academy of Pediatrics guidance to investigate for FMS after the age of one year, unless indicated by other signs of concern, or in the rare case of a late diagnosis [13]. General consensus among experts in the field is that FMS is a concern in the first 2–3 years of life, [6, 14, 15] and that it is a complication that may worsen or improve over time, warranting close monitoring [6, 16]. There is guidance that MRI should be undertaken as indicated throughout childhood [12, 13]. implying that the risk of FMS is not limited to infancy.

In a retrospective review of the routine scans of 36 infants with achondroplasia, only two showed no clinical signs of narrowing of the foramen magnum, implying that there is some degree of narrowing in the majority of infants, with serious narrowing occurring in up to 50% [17].

Cervicomedullary decompression is the standard intervention for foramen magnum stenosis, [6, 14] however, reported rates of the procedure vary widely [6, 11, 15, 18] and it is unclear how many infants will require the intervention and at what age [14] In a study from Cheung et al. assessing the incidence and severity of FMS using the Achondroplasia Foramen Magnum Score, 25% of infants (9/36) required neurosurgery [17]. Decompression surgery is invasive and carries risk, so the overdiagnosis of this complication without guidance from specialists in the management of foramen magnum stenosis in achondroplasia can be problematic and can lead to unnecessary surgery and associated complications [17].

### The European Achondroplasia Forum

The European Achondroplasia Forum (EAF) is a network of specialists in achondroplasia management and patient advocates working in collaboration, with the overall aim of improving care for individuals with achondroplasia. Led by a Steering Committee comprised of clinical geneticists, paediatric endocrinologists, orthopaedic surgeons, paediatricians, a specialist in adult care, a neuropaediatrician, a sleep specialist, a neurosurgeon and a patient advocate, and representing 10 countries from across Europe and the Middle East, the EAF is representative of the achondroplasia clinical community in Europe.

### Aims of developing guiding principles for the detection and management of FMS

The natural history of achondroplasia in infancy and childhood is well documented in the literature [2, 3]. Both the EAF Guiding Principles for Management [16] and the International Consensus Statement Group [12] advocate for close monitoring in the first 2 years of life to enable the provision of anticipatory and timely intervention. While there is agreement on the need for monitoring of foramen magnum stenosis, there is no clear guidance in the literature on the timing of regular monitoring, which assessments should be carried out and when regular screening for FMS should be ceased. The EAF aimed to achieve consensus on these factors, in a bid to provide a standardised best practice protocol for the detection of foramen magnum stenosis.

### Developing guiding principles for detection and management of FMS

A literature search was carried out and the EAF Steering Committee was invited to submit suggestions for guiding principles to address screening for FMS, indicators for intervention, the decision-making process to proceed with decompression, and management of untreated FMS in those who may not have been part of a routine screening programme. Five guiding principles were drafted for discussion at an EAF open workshop; an additional principle was added during the discussion. Attendees were invited to attend the workshop via the EAF website, mailing list and promotion through the Steering Committee and their network of contacts. The workshop was held virtually. The participants in the workshop discussed the draft guiding principles, assessed current practices, and scrutinised the guiding principles to improve and standardise the detection and management of FMS in individuals with achondroplasia.

The EAF open workshop was attended by 47 HCPs and five patient representatives from 17 countries worldwide. Each guiding principle was presented and scrutinised for content, wording, support from the literature or collective expert opinion. During discussion, it was established that an additional principle should be included to specifically cover MRI screening for FMS. Following discussion with the wider group, a draft principle was developed by the lead author (MI) and presented. Live anonymous voting was held to establish whether the six guiding principles were agreed, and the level of agreement with each. The voting process adhered to the EAF Standard Operating Procedure for Developing Guiding Principles/Recommendations, [19] in which a majority of 75% in favour is needed to pass the principle at the first vote. Should 75% not be reached, the text was revised, and a subsequent vote held requiring 70% in favour to pass. Should 70% not be reached, text was revised, and a subsequent vote held requiring a majority of 66% in favour to pass, or the principle was rejected. On reaching the mandated % in favour to pass a proposal, anonymous voting was held on the level of agreement, ranging from 1, no agreement at all, to 10, strong agreement.

The agreed guiding principles are outlined in Table 1. The guiding principles covered (A) the routine clinical monitoring of infants and young children for FMS to the age of 3 years, (B) the timing of routine MRI screening, (C) the referral of suspected FMS to a neurosurgical specialist in achondroplasia when signs of compression are identified, (D) the combination of assessments that should be evaluated by a neurosurgical specialist to inform the decision to decompress the foramen magnum, (E) the joint decision making process to proceed with decompression, and (F) the management of older children in whom previously undetected FMS is identified. After scrutiny and some amendments to the wording, all six guiding principles achieved the ≥ 75% majority needed to pass the principle (range 89–100%), with high levels of agreement (range 7.6–8.9).

**Table 1:** EAF guiding principles for the detection and management of foramen magnum stenosis

Item	Guiding Principle	Vote (%)	Level of agreement (mean; range)
A	All infants should be monitored clinically for foramen magnum stenosis every 3–4 months from birth to the age of 1 year, thereafter every 3–6 months until the age of 3 years. After the age of 3 years, monitoring for foramen magnum stenosis should be based on individual need and local protocols	100	8.4 (4–10)
B	MRI imaging should be undertaken as routine monitoring for foramen magnum stenosis at 3–6 months of age and repeated according to findings in other routine assessments	89	7.9 (2–10)
C	Where signs of compression are observed on screening, infants should be referred as soon as possible to a neurosurgery specialist in a centre experienced in the management of achondroplasia	97	7.6 (1–10)
D	The decision to decompress the foramen magnum should be made using a combination of clinical, neurological and imaging assessments, evaluated by a neurosurgical specialist experienced in the management of achondroplasia	93	8.9 (5–10)
E	The decision to proceed to decompression of the foramen magnum should be made jointly by a neurosurgeon experienced in achondroplasia, the individual’s family and the wider multidisciplinary team	96	8.4 (2–10)
F	Older children and adults with previously undetected foramen magnum stenosis should be managed on an individual basis by the multidisciplinary team	96	8.3 (5–10)

A. ***All infants should be monitored clinically for foramen magnum stenosis every 3–4 months from birth to the age of 1 year, thereafter every 3–6 months until the age of 3 years. After the age of 3 years, monitoring for foramen magnum stenosis should be based on individual need and local protocols***.

A lack of clarity was identified around the word “monitored”, with some people interpreting this as monitoring by MRI, and some as clinical monitoring. The possibility of MRI every 2–3 months was considered excessive by both HCPs and PAG representatives, with considerable burden and stress on the families in scheduling an MRI, waiting for an appointment, potential use of general anaesthetic in infants, and subsequently waiting for the results. This burden is increased if the family are required to travel a large distance to attend a specialist centre. While clinical monitoring can identify indicators of FMS it was strongly advocated that MRI is a vital component in evaluating the presence and extent of FMS. Imaging was excluded from this principle to avoid any confusion that may arise around the word “monitoring”. A revision to the original wording of the principle specifically indicated clinical monitoring. A new principle was drafted to address the MRI evaluation of FMS (see principle B below).

The American Academy of Pediatrics suggest that infants with achondroplasia be assessed for craniocervical junction risk as soon as they receive a diagnosis, with assessments including taking of neurologic history and examination, polysomnography (PSG), and imaging [13]. There are a wide number of assessments that can be used as part of coordinated monitoring for FMS: clinical examination for assessment of tonus, clonus, asymmetry, including early hand preference, head circumference, developmental delays, and general well-being; [4] sleep studies to assess for central and obstructive sleep apnoea, although the link between results of PSG and presence of FMS is unclear; [20, 21] somatosensory evoked potentials to evaluate sensory nerve response which can be indicative of FMS; [5] and regular neurological evaluation [12].

White et al. recommend that comprehensive history and physical examination are carried out every 2 months as part of screening for FMS, [4] while Savarirayan et al. recommend regular evaluation every 2–4 months initially, then every 3–6 months depending on medical concerns [12]. It was agreed during the EAF workshop that clinically monitoring for FMS is feasible at a frequency of every 3–4 months. Indeed, it was suggested that infants without achondroplasia are often reviewed by an HCP every 3–4 months in their first year of life, so assessing for indicators of FMS at this frequency adds no extra burden. Evidence and natural history suggest that the risk of severe complications from cervicomedullary compression is greatest in the first 2 years of life, and that the risk decreases as the child ages. Savarirayan et al. recommend that close clinical monitoring is maintained at least to the age of 3 years [12]. As such, the EAF propose monitoring every 3–4 months during the first year, then every 3–6 months to the age of 3 years.

B. ***MRI imaging should be undertaken as routine monitoring for foramen magnum stenosis at 3–6 months of age and repeated according to findings in other routine assessments***.

While regular clinical evaluations are a key component of monitoring for FMS, and can provide vital clues to a potential problem, there are data to suggest that clinical examination alone may not be sufficient to detect FMS [6, 17]. Cheung et al. found that of 36 infants screened using MRI, only two (5.6%) had no evidence of FMS [17]. In addition, only two had abnormal neurological findings; it was found that cardiorespiratory sleep studies and clinical examination would have failed to detect five infants (27%) with the most severe compression (Achondroplasia Foramen Magnum Score [AFMS] 3–4) [17]. Indeed, 18 of 58 patients (31%) screened in another study had severe cranial stenosis using a scoring system from 0 to 4 [5].

An international consensus states that *MRI is the preferred imaging modality to investigate cervicomedullary compression in infants and children with achondroplasia*, [12] and many centres now routinely screen with MRI during the first year of life. However, there is no universally agreed standard of screening for foramen magnum stenosis, [6] and consensus statements vary in relation to when to screen [4, 12].

MRI is a vital component in the assessment of infants for FMS and screening should be part of routine monitoring. The American Academy of Pediatrics propose the inclusion of MRI as part of a combination of assessments including neurologic history, clinical examination, and PSG [13]. This approach enables the capturing of all potential indicators for FMS, including those that may only be detected on MRI.

There is little in the literature regarding the optimal images to best determine the extent of FMS; which precise images are needed, and in what positions. Systematic sagittal T2 images, as well as axial/transverse images may be preferable, although these may not be available in all centres/countries [20]. Positioning of infants during MRI to capture clear images may be achieved by administering a general anaesthetic, however, the use of general anaesthesia can be a concern for some families (it should be noted that the use of anaesthesia in this instance should be carried out by anaesthetists experienced in achondroplasia). The alternative option is sleep deprivation followed by the ‘feed and wrap’ technique, which is potentially feasible up to the age of 6 months [17]. There is argument for whether the degradation in images using this technique outweighs the concerns for the use of general anaesthetic.

There are scoring systems available to support the interpretation of MRI imaging which categorise what is observed on MRI [5, 17, 22]. Interpretation of MRIs should be made by someone specialised in achondroplasia alongside clinical findings. Technical fine measurements on MRI of posterior and cranial fossa, supratentorial ventricular volume and cranio-cervical junction can also be beneficial to establishing a full understanding of the MRI results. Calandrelli et al. demonstrated a relationship between ventriculomegaly and shortening of the clivus, in addition to premature closure of the posterior cranial fossa synchondroses and shape and direction of growth of the supraoccipital bone, contributing to reduction of the foramen magnum [23].

The optimal timing of MRI is also unclear from the literature and there are questions as to whether an MRI early in life in addition to one slightly later may be beneficial as a routine procedure to monitor change in the foramen magnum. There is concern that if MRI is carried out too early in an infant’s life the maximum extent of stenosis will not be observed, as it can progress over time. As mentioned above, routine, repeated MRIs during the first year were considered burdensome to families, so the consensus was that one routine MRI should be undertaken at 3–6 months of age, with repeat MRIs taken when indicated by findings from other assessments, as recommended in the literature [4, 12].

C. ***Where signs of compression are observed on screening, infants should be referred as soon as possible to a neurosurgery specialist in a centre experienced in the management of achondroplasia***.

When signs of compression are observed, infants should be referred to neurosurgical colleagues with experience in the management of achondroplasia. It was agreed that clinicians in primary or secondary care, and general neurosurgeons would not have the knowledge of achondroplasia required to accurately evaluate any signs of compression, or to plan the next stages of management. There are concerns that interpretation of MRI and clinical indicators of compression by those not experienced in achondroplasia may lead to overtreating and unnecessary procedures.

Signs of compression should then be assessed by a neurosurgeon experienced in the management of achondroplasia [12, 13]. Ensuring that infants are seen by the right people, in the right environment is crucial to enable optimal management.

A range of assessments are necessary to indicate the need for intervention in an infant with FMS, including those outlined above (clinical, neurological, MRI, PSG). The signs of compression on clinical examination that should raise concern and prompt referral to a neurosurgeon specialist in a centre experienced in the management of achondroplasia include hypotonia, hyperreflexia and sustained ankle clonus, asymmetric reflexes, rapid head growth and significant developmental delay [13] (Table 2). Apnoea and hypopnea can be a sign for concern, as can hypoxic episodes with oxygen desaturation. Interpretation of PSG data by a sleep specialist, experienced in achondroplasia is key.

**Table 2:** Factors that may indicate cord compression that should trigger consideration of referral to a neurosurgery specialist experienced in the management of achondroplasia

Type of assessment	Assessment
Neurological	• Hyperreflexia or persistent clonus • Asymmetric reflexes, early hand preference • Increased head circumference, beyond achondroplasia-specific growth charts • Hypotonia • Irritability • Developmental delay, beyond achondroplasia-specific developmental charts
Neuroimaging	• Evidence of cord compression on MRI
Respiratory	• Evidence from polysomnography of central apnoea

The literature recommends repeat MRI when indicated by other assessments [4, 12, 13]. MRI scoring systems can be beneficial, although expert interpretation in the context of other results is still required. For example, a score of AFMS 4 is a clear indicator for intervention, whereas there is a degree of subtlety in AFMS 2–3b which require consideration as part of a breadth of investigations [17]. All signs of compression need to be carefully assessed by a neurosurgical specialist, in the context of the wider clinical picture to enable accurate evaluation.

D. ***The decision to decompress the foramen magnum should be made using a combination of clinical, neurological and imaging assessments, evaluated by a neurosurgical specialist experienced in the management of achondroplasia***.

In alignment with Principle C, the clinician evaluating assessments to inform decision making for decompression should be a neurosurgical specialist. It was emphasised, in line with international guidance, [12] that the neurosurgeon carrying out foramen magnum decompression must be experienced in performing this procedure in paediatric patients with achondroplasia.

Rates of cervicomedullary decompression vary widely between centres. Analysis of a US database found a range of decompression rates from 4.6 to 43% [14]. Some guidance is given in the literature, but there is no clear criteria for when cervicomedullary decompression is indicated [6]. Among other indications, White et al. states that decompression is indicated in the presence of significant stenosis and correlative changes in polysomnography and/or pathological neurologic examination, as well as in MRI-defined FMS in instances where there are spinal cord changes [4]. This is echoed in the protocol adopted by Cheung et al. when assessing the AFMS, [17] while Savarirayan et al. propose a wider indication of symptomatic children with cervicomedullary compression, with or without MRI signal change in the spinal cord [12]. What is clear from the literature is that there are many instances in which decompression may be indicated, and that each situation must be assessed on an individual basis.

The decision to proceed to decompression is complex and requires a combination of multidisciplinary assessments to ensure an accurate evaluation of the indicators, including clinical and neurological evaluation, MRI and PSG [4, 12]. No single indicator for decompression surgery should be used in isolation to make the decision to proceed, [4, 13] and the absence of specific indicators should not rule out surgical intervention [12, 17]. Use of scoring systems including the AFMS for MRI results, [17] and the adjusted developmental milestones tool for clinical assessment can be helpful in the decision-making process [24]. There are many permutations to each individual situation that need to be considered prior to proceeding to decompression.

E. ***The decision to proceed to decompression of the foramen magnum should be made jointly by a neurosurgeon experienced in achondroplasia, the individual’s family and the wider multidisciplinary team (MDT)***.

This principle was widely accepted, with little requirement for discussion and no changes to the wording. As has been discussed above, and is supported by the literature, a neurosurgical team experienced in decompression of the foramen magnum in achondroplasia are vital to the decision-making process whether to proceed to surgery, [12] however, this decision cannot be taken in isolation. Achondroplasia is a lifelong condition requiring lifelong management by an experienced MDT, and decisions around management should be made in the MDT setting jointly with the person with achondroplasia and/or their family [16]. Evidence suggests that patient/family involvement in decision-making can improve patient satisfaction, confidence and clinical outcomes [25–27]. As the patients are infants, the involvement of the family in understanding the reasons for decompression and in the decision-making process is crucial.

F. ***Older children and adults with previously undetected foramen magnum stenosis should be managed on an individual basis by the MDT***.

Foramen magnum stenosis in older children and adults is rare; it is not known how many individuals have previously undetected or untreated FMS. As such, it was agreed that local protocols are unlikely to exist, and that it was preferable to direct individuals to the MDT, rather than suggest centres develop protocols for this situation.

While recent advances in screening protocols will help to identify FMS in those who have access to screening, there will be children who were not routinely screened in previous years. MRI is often requested before a surgery, such as orthopaedic or ENT procedures. It was noted that MRI scans done later in life, in those who had never undergone decompression surgery, may show myelopathy in the presence of a more spacious foramen magnum. This suggests that the foramen magnum was more narrow relative to the cord in the past to a degree that may have resulted in significant cord compression. However, more data are needed to clarify the relationship between myelopathy and foramen magnum stenosis in this uncommon situation. One Steering Committee member with extensive experience of reviewing full spinal MRIs prior to limb lengthening, commented that FMS in 7–10-year-olds is extremely rare, however, several clinicians were aware of individuals with untreated foramen magnum stenosis. Interestingly, different reasons were provided as to why these cases had not been treated, including delayed diagnosis and/or lack of screening at a previous centre, presentation before screening was routinely undertaken, signal intensity abnormalities of the cervical spine but lack of other symptoms, or that policies were not in place for this specific issue.

There is limited data on FMS in older children, and more is needed. MRI scans across age groups would be beneficial to define different growth patterns of the foramen magnum, identify key signs for concern and enable risk stratification. With children now in clinical trials and longer-term data available, there is potential to capture natural history data while they are receiving routine MRIs as part of a trial protocol [28]. Understanding the development of FMS over time in children with no clinical complications, may help to identify which children should be routinely screened for longer than 2–3 years.

Other gaps in our knowledge are the risk factors and red flags for FMS after the age of 2–3 years. It is recognised that myelopathy may be evident on MRI scans in older children in the absence of clinical signs of FMS, [5] however, it is unclear whether myelopathy indicates the need for decompression in older children if there is no recent aggravation on neurological clinical signs. More data is needed on this subject to enable detailed recommendations to be made; a consolidation of the cases and clinical experience would also provide some insights into this rare situation.

Recent international consensus recommendations do not contain any specific recommendations for children who have stenosis but were not treated in the past. This underlines the need for guidance from the EAF. It was widely agreed that while no recommendations exist for previously undetected FMS in older children, neurological signs of concern should indicate further investigation. As these instances are likely to be rare, each person presenting with previously undetected FMS should be managed on an individual basis by an MDT, per EAF guiding principles [16].

## Conclusions

In developing the guiding principles for the detection and management of foramen magnum stenosis, the EAF aimed to provide clarity to existing recommendations on monitoring protocols, appropriate assessments, and actions on identification of indicators of concern in relation to foramen magnum stenosis either in infants, or in the rare cases presenting after 2 years of age. All those involved in developing the guiding principles and in the consensus gathering process are highly knowledgeable about achondroplasia and the management of its complications, including foramen magnum stenosis, with specialists in European reference centres in attendance. While these principles may be relevant to other conditions in which foramen magnum stenosis is a consideration, they were developed specifically for the occurrence in individuals with achondroplasia. The principles were developed with the aim of providing guidance that can be applied in all healthcare systems, alongside existing recommendations and country, regional and centre protocols. It is hoped that by improving clarity around the detection and management of FMS, infants and young children will receive optimal monitoring for this potentially life-threatening complication ensuring those who need cervicomedullary decompression receive it in a timely manner, and that the issue of overtreating can be mitigated. These principles will be revisited in future to ensure they remain relevant among a changing clinical and treatment landscape.

## Data Availability

Not applicable.

## Declarations

**Ethics approval and consent to participate**.

**Consent for publication**.

## List of abbreviations

AFMS: Achondroplasia Foramen Magnum Score
EAF: European Achondroplasia Forum
FMS: Foramen Magnum Stenosis
HCP: Healthcare Professional
MDT: Multidisciplinary team
MRI: Magnetic Resonance Imaging
PSG: Polysomnography
